# Impact of Temperature and Atmospheric Pressure on Hospitalizations of Patients Presenting With Acute Coronary Syndrome

**DOI:** 10.7759/cureus.54833

**Published:** 2024-02-24

**Authors:** Naltin Shuka, Andri Cabeli, Leonard Simoni, Mirald Gina, Ledjana Kondi, Edvin Dado

**Affiliations:** 1 Cardiovascular Medicine, University Hospital Center "Mother Teresa", Tirana, ALB; 2 Physiology, University of Medicine, Tirana, Tirana, ALB; 3 Cardiovascular Disease, University Hospital Center "Mother Teresa", Tirana, ALB; 4 Cardiovascular Disease, University of Medicine, Tirana, Tirana, ALB

**Keywords:** acute coronary syndrome (acs), daily changes, season changes, atmospheric pressure, air temperature

## Abstract

Aim: This study aims to investigate the impact of temperature and atmospheric pressure on hospitalizations of patients with acute coronary syndrome (ACS).

Materials and methods: This is a retrospective, observational, analytical study conducted in a single center, University Hospital Center "Mother Teresa," Tirana, Albania, in the period January-December 2018. This study included 1,165 patients with ACS, who performed urgent coronary angiography, from January 2018 to December 2018. Patients were diagnosed with ACS based on clinical and examination findings. The data were collected retrospectively using patient files. Baseline demographic, clinical, and procedural characteristics were collected. Data on atmospheric parameters, measured at the weather monitoring station, were obtained from the National Meteorological Service database. Measurements from the meteorological service provided values ​​for each parameter: average daily temperature and atmospheric pressure in each country district. Atmospheric data measurements were taken for the day under review. The number of inhabitants for the respective districts is taken from the National Institute of Statistics (INSTAT).

Results: The study involved 1,165 patients, with a mean age of 63.1 years, ranging from 27 years to 89 years old. The majority of patients (78.6%) were male, while 21.4% were female. A statistically significant relationship was observed between seasonal changes in temperature and atmospheric pressure concerning the number of cases with ACS; the autumn season prevails with 27.9% of the total cases, followed by the spring season with 25.6%, the summer season with 24.2%, and winter season with 22.3% (p = 0.04). Additionally, significant changes in the average monthly values ​​of temperature and atmospheric pressure were accompanied by a statistically significant increase in the number of cases as occurred in March-April and October-November (p ≤ 0.05). Most cases in the cold period (November-March) occurred on days with a change in temperature or atmospheric pressure with a statistically significant value of p < 0.05.

Conclusion: An important relationship between seasonal, monthly, and daily changes in temperature and atmospheric pressure concerning the frequency of cases with ACS was observed.

## Introduction

Meteorological conditions are studied as an important risk factor for acute cardiovascular events. Humidity, barometric pressure, wind speed, and temperature changes are closely related to acute coronary syndrome (ACS) [1]. ACS remains one of the most common health problems in the world and the leading cause of death. Previous studies have identified several factors and triggers associated with ACS. Moreover, over the past few decades, growing epidemiological and clinical evidence has led to heightened concerns about the potential short- and long-term effects of the environment on cardiovascular health, especially on ACS. Although the risk of climate and environmental factors is not so evident compared with the effect of well-established risk factors, the public health relevance is considerable, as environmental factors impact hundreds of millions of people. The Lancet Commission on Health and Climate Change has declared that the biggest health challenge in the 21st century is climate change [2]. Unfavorable atmospheric situations caused by climate change are predicted to increase the number of acute cardiovascular diseases (ACVDs) mainly. Thus, a better understanding of atmospheric parameters can help establish new cardiovascular prevention strategies against them. Several studies have examined the cardiovascular effects of atmospheric parameters as separate factors, such as temperature, and atmospheric pressure; however, few have investigated atmospheric parameters’ joint effects. The incidence of temperature-related mortality is attributed more to cold than extreme heat [3]. The impact of cold temperatures on myocardial infarction (MI) hospitalizations has been well-described in the literature, whereas the impact of heat on acute MI has been less robust. The relationship between heat and the heart is an area of ongoing research. However, scholars have emphasized that heat, especially extreme heat, is significantly correlated with the rate of death attributable to CVD [4,5]. The consequences of atmospheric pressure on cardiovascular diseases have been studied less frequently probably because most studies analyzed only monthly or seasonal variations of event rates. Indeed, the variability of monthly atmospheric pressure is weaker than daily variations, often leading to inconclusive results [6]. This study is aimed to investigate the impact of temperature and atmospheric pressure on the hospitalizations of patients with ACS.

## Materials and methods

Study design and population

This is a retrospective, observational, analytical study conducted in a single center - University Hospital Center "Mother Teresa," in Tirana, Albania, during the period January-December 2018. The study complies with the Declaration of Helsinki and was approved by the Local Ethics Committee of the hospital. Informed consent was taken from every patient.

The study included 1,165 patients with ACS, hospitalized in the hospital who performed emergency coronary angiography. Eighty-five patients initially diagnosed with ACS, a diagnosis that was not confirmed at the discharge of the patient from the hospital, were excluded from the study. In all these cases, angiography had resulted in no coronary artery stenosis.

Data collection

The data were collected retrospectively using patient records from archived files at the Statistics Center. Baseline demographic, clinical, and angiographic characteristics were collected. Data on atmospheric parameters, measured at the weather monitoring station, were obtained from the National Meteorological Service database. Measurements from the meteorological service provided values for each parameter: average daily temperature and atmospheric pressure in each country district. Atmospheric data measurements were taken for the day under review. The number of inhabitants for the respective districts is taken from the National Institute of Statistics (INSTAT). The change of temperature/pressure was calculated as the difference of 48 hours before ACS and that on the day of ACS and temperature change considered the difference values of ≥ 4 °C and 4 °C, based on the histogram of the distribution of temperature difference values. The pressure difference was considered to be the difference values of ≥ 4 Pa and ≤ -4 Pa, based on the distribution histogram of the pressure difference values.

Statistical analysis

The statistical program Statistical Product and Service Solutions (SPSS, version 20.0; IBM SPSS Statistics for Windows, Armonk, NY) was used for data analysis. The categorical variables were presented according to their absolute and relative frequency, expressed as a percentage, and the statistical test hi-square (χ2) was used to compare them. Continuous data were presented with a mean (M) and standard deviation (SD). The Student t-test was used for two independent samples, and the t-test was used for two pairs of samples; one-way analysis of variance (ANOVA) was used to compare the mean of continuous variables. Pearson correlation was used to estimate the relationship between the continuous variables. Receiver operating characteristic (ROC) curves were used, and the following was determined: sensitivity and specificity of continuous variables. Univariate and multivariate logistic regression analysis checking for possible confounders was used to assess the impact of the season on ACS and STEMI. The relative risk of daily ACS is calculated through multivariate analysis or the Poisson model. Statistical significance is defined for the p value ≤ 0.05. Statistical tests are two-sided, and tables and graphs were used to visualize the data.

## Results

The study involved 1,165 patients with a clinical diagnosis of ACS. The majority of patients (78.6%) are male, while 21.4% are female (p < 0.01). For the study, we classified the patients into three age groups. The study showed that the most affected age group is those over 55 years old with 919 patients, followed by those 45-54 years old with 190 patients. The least affected age groups were those under 44 years old with 56 patients (p < 0.01). In the distribution of cases by month, it seems that the largest number of cases with ACS occurred in October at 10.4%, while the month with the lowest number was January with 6.6%. No significant differences were seen in the monthly distribution of cases with ACS (p = 0.1). April, August, and October had the largest number of cases with a statistically significant difference compared to the other months (p ≤ 0.05). In the distribution of cases with ACS by season, it seems that ACS prevails in the autumn season, with 27.9% of the total cases, followed by the spring season (with 25.6%), the summer season (with 24.2%), and the winter season (with 22.3%; p = 0.04) (Table 1).

**Table 1:** Distribution of patients diagnosed with ACS by gender, age group, months, and seasons SD - Standard deviation

Variable	N	%	P value
Sex			<0.01
Female	249	21.4	
Male	916	78.6	
Age mean (SD)	63.1 (10.5)		
Age			<0.01
≤44	56	4.8	
45-54	190	16.3	
≥55	919	78.9	
Month			0.1
January	77	6.6	
February	85	7.3	
March	93	8.0	
April	105	9.0	
May	100	8.6	
June	84	7.2	
July	91	7.8	
August	107	9.2	
September	102	8.8	
October	121	10.4	
November	102	8.8	
December	98	8.4	
Season			
Spring	298	25.6	0.04
Summer	282	24.2	
Autumn	325	27.9	
Winter	260	22.3	

**Figure 1:**
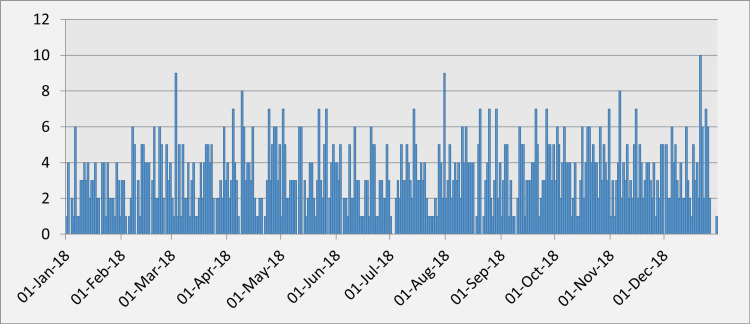
Distribution of cases by days

Regarding the daily frequency, the average number of cases with ACS is 3.2 and varies from 0 to 10 cases per day. The largest number of patients with ACS in one day was around 9-10 in March, August, and December. October is the month with the most days with three or more cases per day with ACS (Table 2, Figure 1).

**Table 2:** Number of cases of ACS by months and days

Month	Day with new cases			Days
	0-1	2-3	>3	
January	9	13	9	31
February	7	9	12	28
March	7	12	12	31
April	5	11	13	30
May	6	13	12	31
June	8	14	8	30
July	8	13	10	31
August	5	10	16	31
September	7	10	13	30
October	3	8	20	31
November	3	14	13	30
December	8	12	12	31
	76 (20.8%)	139 (38.1%)	150 (41.1%)	365

It is observed that all three diagnoses are encountered, during all months of the year, but with a statistically significant predominance of STEMI (76.7%), followed by unstable angina (UA, 14.5%) and NSTEMI (8.8%; p < 0.01), a result that corresponds to the nature of hospitalizations in the hospital.

**Figure 2:**
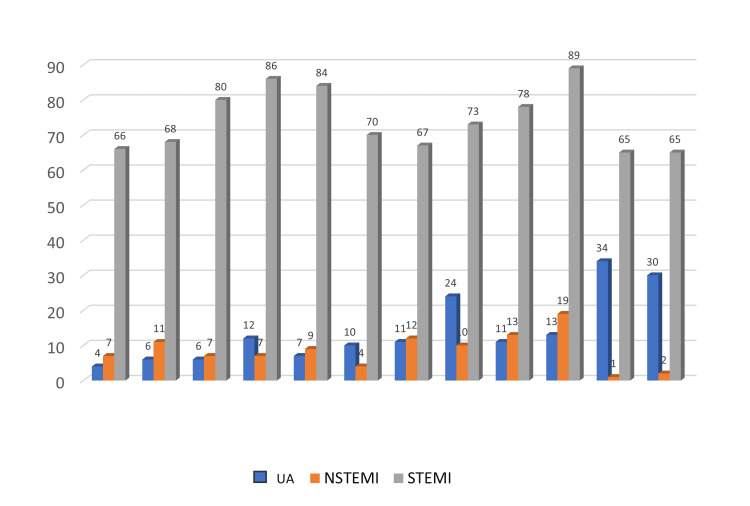
Distribution of cases according to diagnosis and months UA - Unstable angina, NSTEMI - Non-ST elevation myocardial infarction, STEMI - ST elevation myocardial infarction

Analyzing the relative frequency, it is observed that STEMI is encountered almost uniformly throughout the months of the year, with a slight increase in October, while NSTEMI and UA have their peaks in October (18.6%) and November (20.2%), respectively (Figure 2).

It is observed that the peak of anterior STEMI is in October and in April, with 40 cases in each month. The peak of inferior STEMI is in March and September, with 49 and 42 cases, respectively. The peak of lateral STEMI was recorded in March with six cases, followed by September with five cases. In total, the diagnosis of STEMI is encountered more during the spring season (27.9%), followed by the autumn season (26%), summer season (23.5%), and winter season (22.6%), without statistically significant changes between them (p = 0.6).

Regarding the age groups ≤ 44 years and those between 45 and 54 years, there was a statistically significant impact of the temperature change from 48 hours to 24 hours before the occurrence of ACS (p < 0.01 and p = 0.03, respectively), but not for the age group ≥ 55 years. This index was not statistically significant regarding sex: male or female.

Regarding the diagnosis, there is a statistically significant change in temperature from 48 hours to the day of the event for UA (p = 0.02) and STEMI (p < 0.01), but not for NSTEMI. There is a statistically significant change in temperature from 48 hours to 24 hours on the day of the event in patients with inferior STEMI, with a value of p = 0.04, but not the other types of STEMI. A significant temperature change was found from 48 hours to 24 hours and the day of the event for two-vessel disease cases with a p value < 0.01 (Table 3).

**Table 3:** Clinical characteristics of patients by temperature T - temperature, ACS - Acute coronary syndrome, NSTEMI - Non-ST elevation myocardial infarction, STEMI - ST elevation myocardial infarction

Variable	T48h before ACS	T24h before ACS	T day ACS	P
Age				
≤44	15.8 (7.7)	16.5 (7.6)	17.0 (7.4)	<0.01
45-54	17.7 (7.0)	17.9 (6.9)	18.1 (6.7)	0.03
≥55	18.1 (6.9)	18.2 (6.9)	18.2 (7.0)	0.7
Sex				
Female	17.4 (7.2)	17.5 (7.2)	17.5 (7.2)	0.5
Male	18.1 (6.9)	18.2 (6.9)	18.3 (6.9)	0.08
Diagnosis				
Unstable Angina	17.9 (7.1)	17.8 (7.3)	17.5 (7.5)	0.02
NSTEMI	19.1 (7.0)	19.1 (6.9)	19.4 (3.3)	0.3
STEMI	17.8 (7.0)	17.9 (6.9)	18.1 (7.0)	<0.01
Concomitant Diseases				
High Blood Pressure	18.9 (6.8)	19.1 (6.8)	19.2 (6.8)	0.3
Diabetes Mellitus	18.7 (6.9)	18.8 (6.8)	18.8 (6.7)	0.2
Coronary Angiography				
1-vessel disease	18.7 (7.0)	18.8 (6.9)	18.9 (7.1)	0.2
2-vessel disease	18.8 (6.8)	19.1 (6.7)	18.1 (6.7)	<0.01
3-vessel disease	19.9 (6.4)	19.8 (6.6)	20.0 (6.4)	0.4
STEMI type				
Anterior	17.5 (6.9)	17.6 (6.9)	17.7 (6.9)	0.8
Inferior	18.0 (6.9)	18.2 (6.9)	18.3 (7.0)	0.04
Lateral	16.9 (7.4)	17.4 (6.5)	17.2 (7.4)	0.6
Posterior	18.5 (6.9)	18.8 (6.8)	18.2 (7.2)	0.5
Severe Complications	17.7 (6.6)	17.7 (6.7)	17.7 (7.2)	0.8
Death	18.6 (7.8)	18.8 (7.8)	18.8 (7.9)	0.6

A statistically significant change in atmospheric pressure, 48 hours before ACS, 24 hours before ACS, and on the day of the event for the ≤ 44 age group and 45-54 age group was found (p = 0.03 and p = 0.05, respectively), but not for the age group ≥ 55 years. Regarding the diagnosis, a significant change in atmospheric pressure was found from 48 hours to 24 hours before and on the day of ACS, for NSTEMI (p = 0.02), but not for other subtypes of ACS. A statistically significant change of atmospheric pressure was observed from 48 hours to 24 hours before and day of ACS for one-vessel disease and two-vessel disease, with respective values of p = 0.01 and p = 0.05 (Table 4).

**Table 4:** Clinical characteristics of patients according to atmospheric pressure P - Pressure, ACS - Acute coronary syndrome, NSTEMI - Non-ST elevation myocardial infarction, STEMI - ST-elevation myocardial infarction

Variable	P48h before ACS	P24h before ACS	P day ACS	P
Age				
≤44	1014.3 (5.5)	1013.1 (5.1)	1012.2 (5.4)	0.03
45-54	1014.4 (5.9)	1014.1 (6.1)	1013.5 (6.2)	0.05
≥55	1013.8 (5.6)	1013.9 (5.7)	1013.8 (5.7)	0.7
Sex				
Female	1013.9 (5.4)	1013.8 (5.9)	1013.5 (5.8)	0.6
Male	1013.9 (5.8)	1013.9 (5.7)	1013.8 (5.8)	0.6
Diagnosis				
Unstable Angina	1013.9 (5.2)	1013.9 (5.0)	1014.3 (4.8)	0.3
NSTEMI	1014.7 (5.6)	1014.2 (5.6)	1013.3 (6.0)	0.02
STEMI	1013.8 (5.7)	1013.8 (5.9)	1013.6 (5.9)	0.2
Concomitant Diseases				
Hypertension	1013.5 (5.6)	1013.5 (5.6)	1013.4 (5.7)	0.5
Diabetes Mellitus	1013.6 (5.6)	1013.5 (5.7)	1013.5 (5.7)	0.6
Coronary Angiography				
1-vessel disease	1014.1 (5.3)	1013.8 (5.6)	1012.2 (5.4)	0.01
2-vessel disease	1014.1 (5.8)	1014.1 (6.1)	1013.5 (6.2)	0.05
3-vessel disease	1013.9 (5.6)	1013.9 (5.7)	1013.8 (5.7)	0.9
STEMI Type				
Anterior	1014.3 (5.9)	1014.2 (6.0)	1014.1 (5.9)	0.4
Inferior	1013.5 (5.6)	1013.6 (5.8)	1013.4 (5.9)	0.7
Lateral	1014.5 (5.0)	1013.3 (6.2)	1012.6 (6.7)	0.1
Posterior	1011.7 (6.6)	1012.4 (4.6)	1011.9 (5.4)	0.9
Severe Complications	1013.6 (5.3)	1013.8 (5.9)	1013.9 (5.2)	0.6
Death	1012.3 (5.3)	1012.8 (5.2)	1013.9 (4.7)	0.07

**Figure 3:**
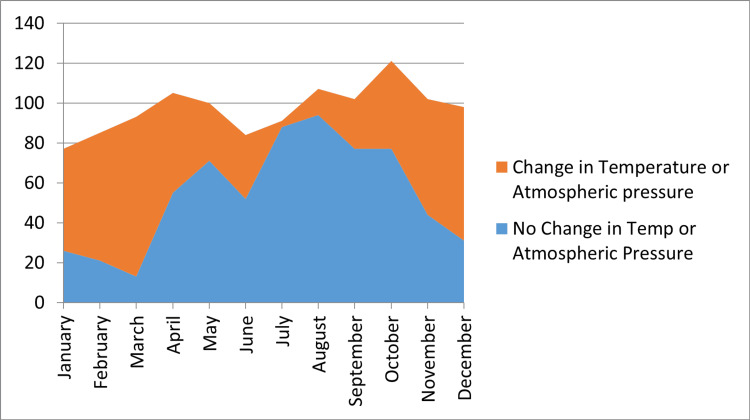
Cases of ACS by months and changes in temperature and atmospheric pressure

It is observed that most of the cases with ACS, in the cold period of the year (November-March), occurred during significant changes in temperature or atmospheric pressure with a p value of < 0.05 (Figure 3).

**Figure 4:**
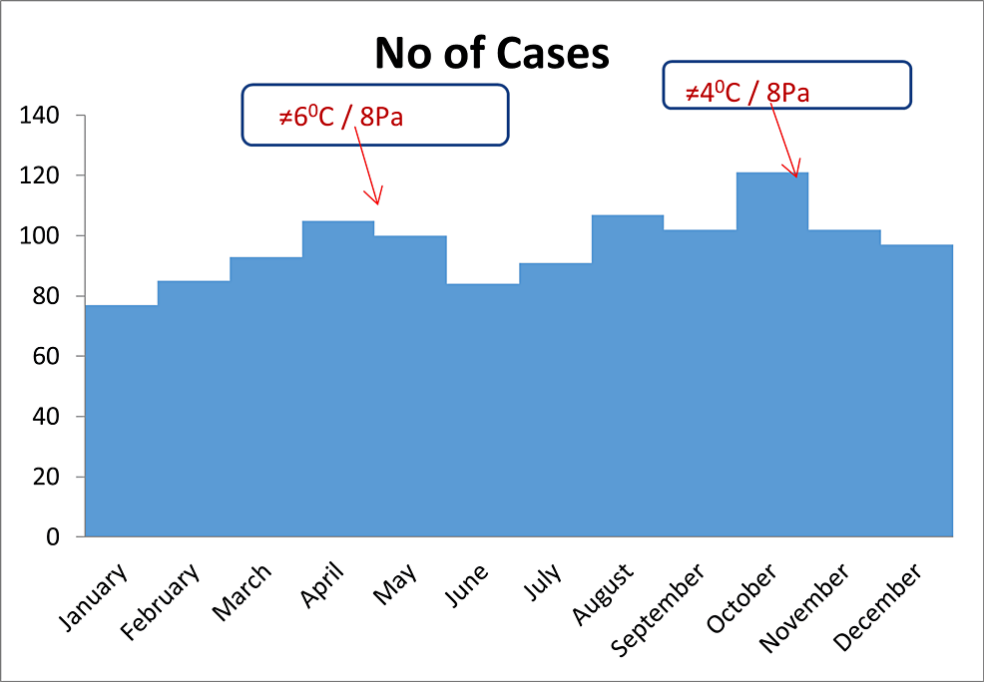
Frequency of ACS by months

The graph below shows the significant impact that has had on the change of the average monthly values of temperature and atmospheric pressure. During the transition from March to April and from September to October, significant changes in temperature and atmospheric pressure were observed, which were associated with a pronounced increase in the number of patients in both cases: in March-April, temperatures varied by 6 °C and pressure by 8 Pa, and in September-October, temperatures varied by 4 °C and pressures by 8 Pa (Figure 4).

**Figure 5:**
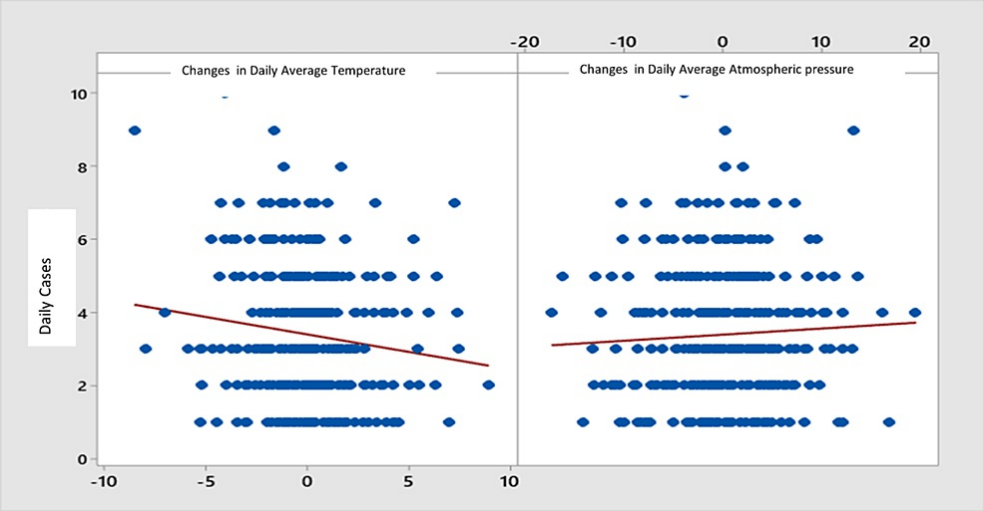
Correlation of ACS with the change in temperature and pressure

A statistically significant correlation was found between the number of daily cases, with the greater temperature change (r = -0.13, p < 0.01), but not with the change in atmospheric pressure (r = 0.05, p = 0.3). The number of cases decreases with increasing temperature and increases with increasing atmospheric pressure (Figure 5).

**Figure 6:**
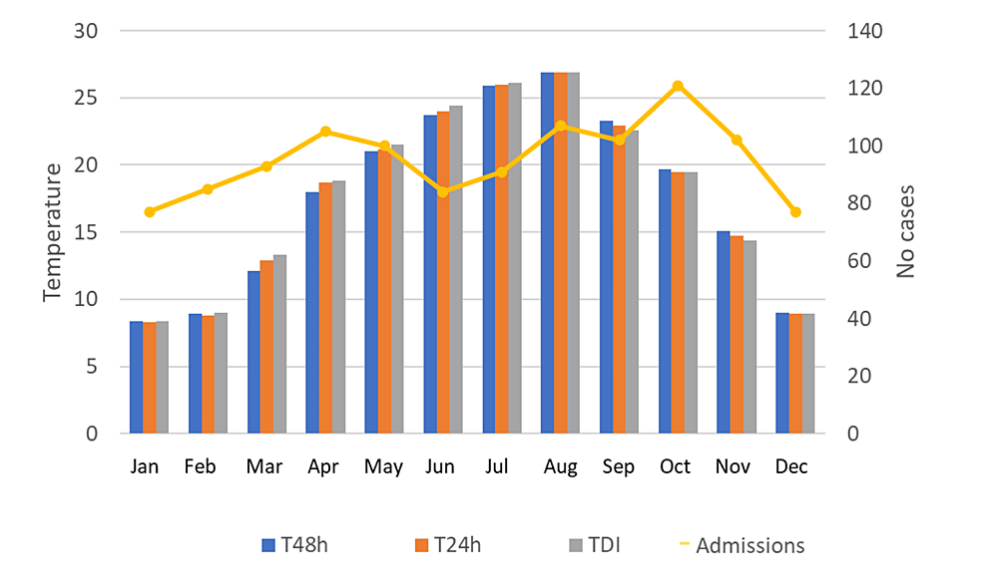
Monthly number of cases of ACS according to average monthly temperatures T48h - Temperature 48 hours before the event, T24h - Temperature 24 hours before the event, TDI - Temperature in the event day

The average monthly temperatures show a steady increase, reaching the peak in August and with a gradual decline until December, while the number of monthly cases represents a significant downward trend in May-June and reaches a peak in October, with a value of p = 0.04 (Figure 6).

**Figure 7:**
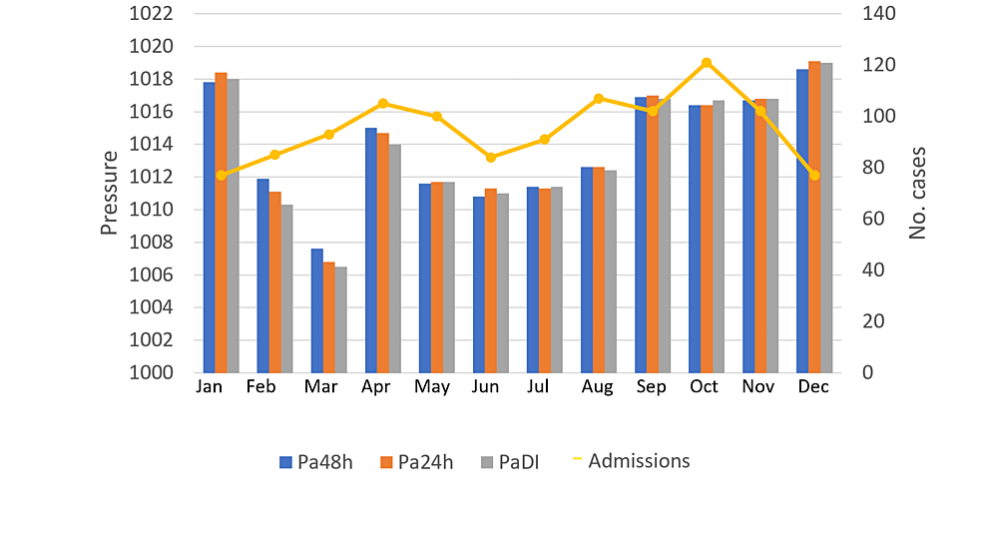
Monthly number of cases with ACS according to average monthly atmospheric pressures Pa48h - Pressure 48 hours before the event, Pa24h - Pressure 24 hours before the event, PaDI - Pressure in event day

The average monthly atmospheric pressure shows significant fluctuations, with a decrease in March, an increase in April, and again a decline that continues until July, followed by an increasing trend from September to December; meanwhile, the number of monthly cases reflects a significant downward trend in the months of May-June and reaches a peak in October (p = 0.04; Figure 7).

In the univariate logistic regression analysis, a significant relationship was found with the occurrence of ACS in April (OR = 2.15, 95%CI = 1.0135-4.59, p = 0.04) and in October (OR = 2.21, 95%CI = 1.0858-4.50, p = 0.03), and regarding the season, ACS occurs more in the spring season (OR = 2.05, 95%CI = 1.0858-4.50, p < 0.01; Table 5).

**Table 5:** Influence of months and seasons on ACS (univariate analysis) OR - Odd ratio, CI - Confidence interval

Variables	OR	95% CI	P
Month			
April	2.15	1.0135-4.59	0.04
October	2.21	1.0858-4.50	0.03
Season			
Spring	2.05	1.38-3.0434	<0.001

In the multivariate analysis of the effect of temperature and pressure on the daily number of ACS, a significant relationship was found only for the temperature 48 hours before the MI (p = 0.02; Table 6).

**Table 6:** Multivariate analysis: relative risk of daily ACS (Poisson model) RR - Relative risk, CI - Confidence interval, T_48h - Temperature 48 h before the day of ACS, T_24h - Temperature 24 h before the day of ACS, T-day of AMI - Temperature in the day of ACS, P_48h - Atmospheric pressure 48 h before the day of ACS, P_ 24h - Atmospheric pressure 24 before the day of ACS, P_day of ACS - Atmospheric pressure in the day of ACS, Daily change T.≥4°C - Daily change of temperature with ≥4°C, Daily change Pa≥4 Pa - Daily change of pressure with ≥4 Pascal, Daily change interaction at T. ≥4 °C & P ≥4Pa - Daily change interaction of temperature with ≥4°C and Daily change of pressure with ≥4 Pascal

Variables	RR	95% CI	P
Temperature			
T_48h	0.89	0.828-0.975	0.02
T_24h	0.99	0.97-1.01	0.5
T_day of ACS	0.99	0.973-1.012	0.5
Daily Change T. ≥4 °C	1.21	0.877-1.651	0.3
Pressure			
P_48	0.98	0.96-1.013	0.4
P_24h	0.99	0.972-1.010	0.4
P_day of ACS	0.99	0.971-1.017	0.6
Daily Change P ≥4 Pa	1.24	0.914-3.24	0.3
Daily change interaction at T. ≥4°C & P ≥4 Pa	1.16	0.828-2.75	0.4

In the multivariate analysis of the effect of temperature and pressure on the daily number of ACS, in patients with hypertension, a significant relationship was found for T_48h (p = 0.01), P_48h (p = 0.03; Table 7). In the multivariate analysis of the effect of temperature and pressure on the daily number of ACS, in patients with DM, a significant relationship was found for P_48h (p = 0.01), P_24h (p < 0.01), and P_day of admission (p < 0.01; Table 7).

**Table 7:** Multivariate analysis: relative risk of daily ACS for patients with HBP or DM (Poisson model) RR - Relative risk, CI - Confidence interval, T_48h - Temperature 48 h before the day of ACS, T_24h - Temperature 24 h before the day of ACS, T-day of AMI - Temperature in the day of ACS, P_48 h - Atmospheric pressure 48 h before the day of ACS, P_ 24h - Atmospheric pressure 24 before the day of ACS, P_day of ACS - Atmospheric pressure in the day of ACS, Daily change T.≥4°C - Daily change of temperature with ≥4°C, Daily change Pa≥4Pa - Daily change of pressure with ≥4 Pascal, Daily change interaction at T. ≥4 °C & P ≥4Pa - Daily change interaction of temperature with ≥4°C and daily change of pressure with ≥4 Pascal

	HBP			Diabetes mellitus		
Variable	RR	95%CI	P	RR	95%CI	P
Temperature						
T_48h	0.89	0.808 to 0.981	0.01	0.87	0.760 to 1.016	0.08
T_24h	1.12	0.983 to 1.282	0.08	1.16	0.952 to 1.414	0.1
T_day of ACS	0.97	0.890 to 1.070	0.6	0.93	0.814 to 1.080	0.3
Daily Change T. ≥4 °C	1.01	0.558 to 1.830	0.9	1.06	0.420 to 2.682	0.8
Pressure						
P_48h	0.94	0.896 to 0.997	0.03	0.90	0.837 to 0.981	0.01
P_24h	1.07	0.96 to 1.153	0.07	1.18	1.064 to 1.314	<0.001
P_ day of ACS	0.95	0.908 to 1.007	0.09	0.88	0.818 to 0.963	<0.001
Daily Change Pa≥4 Pa	0.66	0.305 to 1.442	0.3	1.03	0.338 to 3.163	0.9
Daily change interaction at T. ≥4 °C & P ≥4Pa	1.16	0.481 to 2.795	0.7	0.80	0.220 to 2.948	0.7

## Discussion

The study of the impact of climate and environmental changes on cardiovascular disease has made more evident the lack of formal guidelines and recommendations for this problem. The results of our study contribute to the attitude regarding the impact of atmospheric factors on cardiac health.

Low temperatures cause several pathophysiological changes and can be considered a risk factor for coronary artery disease. At low temperatures, there is an increase in sympathetic nervous activity; the stimulation of cold receptors in the skin increases the levels of plasma catecholamines and vasopressin causing vasoconstriction. As a result, the growth of sympathetic activity can be the reason for the instability of the atherosclerotic plaque and accelerate myocardial ischemia [7]. All these mechanisms contribute to the increase in blood pressure [8]. In addition, a drop in temperature causes an increased diuresis and hemoconcentration, which can result in increased plasma concentrations, coagulation factors, and the number of platelets, which can promote thrombosis. Cold can induce increased levels of inflammatory status and hypercoagulability, with a higher risk of developing thrombogenesis [9]. Cold also favors proatherogenic effects by influencing the level of endothelin-1 and the level of nitrogen monoxide (NO) endothelial [10]. In the cold temperatures of the winter season, levels of more important atherogenic dyslipidemia favor coronary disease [11]. Upper respiratory tract infections, with a higher incidence in the winter season, can increase cardiac output and thus stress on atherosclerotic plaques contributing to their instability and acute cardiovascular accidents [12].

Hot temperatures have similar underlying mechanisms of cardiovascular disease as cold. We also note here the role of dehydration that affects blood concentration, hypercoagulability, sympathetic activation, and inflammatory mediators. Dehydration can promote an increase in heart rate and cardiac metabolic needs, which, in patients with a basic cardiovascular disease, can cause a demand-supply mismatch, causing ischemic events and acute cerebral or cardiovascular events. During extreme heat, increased sweating and evaporation occur. This reaction increases the risk of blood clots, an increase in heart rate, and the risk of destabilization of the coronary atherosclerotic plaque [13].

A weak negative effect of atmospheric pressure (range: 720-750 mbar) on the blood pressure level has been reported in hypertensive patients who did not respond to treatments; this association between atmospheric pressure and a common risk factor of coronary artery disease may offer a clue in our exploration of a biological mechanism underlying the effect of atmospheric pressure on coronary heart disease [14].

Extreme temperature values and temperature changes are contributing factors for ACS. Various studies have emphasized the role of temperature changes, especially between the environment of the house and the external environment, considering these changes more important than the absolute value of the air temperature itself [15]. Numerous studies in elderly patients > 65 years have shown that the elderly population, with a tendency to ischemic disease, is more predisposed to acute cardiovascular events with large changes in temperatures and atmospheric conditions, due to lower thermoregulatory and baroregulation responses [16].

In our study, the distribution of cases of ACS according to the seasons turned out to be higher in autumn, followed by spring, summer, and winter. Some studies have similar results. The seven-year study by Houck et al. [17], in France, highlighted an increasing trend of cases of ACS in January-May, followed by a plateau phase in May-August and an increase in the number of cases in August-December. Our result is also supported by findings of the study in South Korea, by Lee et al. [18], where the highest incidence of cases is seen in the autumn, followed by the spring and winter seasons. There are several other studies, where the incidence of ACS has been higher in the winter season, such as the studies by Sheth et al. [19] and Marchant et al. [20], conducted in Canada and England, respectively, which showed that the incidence and mortality of ACS is higher in the winter season. Another study by Hodzic et al. [21], conducted in Sarajevo, shows that the highest incidence of ACS was recorded in December during the winter season, while the lowest incidence was recorded in March.

The trend in September-December, which is accompanied by a gradual decrease in temperatures and an increase in atmospheric pressure precisely in the autumn season, explains why the incidence of ACS is significantly higher in the autumn season.

STEMI in our study is encountered more during the spring season, followed by the autumn season, summer season, and winter season, but without statistically significant differences between them. This result is similar to the study by Lee et al. [18], in South Korea, which is the only study that has proven the highest incidence of STEMI specifically in the spring season. The study by Leibowitz et al. [22] showed a higher incidence of STEMI in the winter season. While in the studies of Hodzic et al. [21] and Sohrabi et al. [23], conducted in Iran, did not show significant changes in the incidence of STEMI, according to the seasons.

There is a statistically significant effect of the temperature change from T_48h to T-day MI in terms of the occurrence of ACS according to the diagnosis, for UA (p = 0.02) and STEMI (p <0.01). This result is different from that of the study of Lee et al. [18] where the relative risk for ACS, from the difference in temperature from 48 hours before to the day of infarction of 4.5 °C, was higher in the group of patients with NSTEMI.

In the multivariate analysis of the effect of temperature and pressure on the daily number of ACS, a significant relationship was found only for T 48h before infarction (p = 0.02). Numerous studies support the significant role of lowering the temperature in the daily hospitalization of ACS. Such a study is that of Cheng et al. [24], conducted in Brisbane, Australia, who concluded that any drop in temperature by 1 °C (below a reference value of 16 °C) is associated with an increase in risk for ACS by 1.6%, within a day of change, and the effect of this temperature change lasts up to 10 days.

In patients with hypertension, the multivariate analysis of the effect of temperature and pressure on the daily number of ACS shows a significant relationship for both T_48h (p = 0.01) and P_48h (p = 0.03).

Beyond the well-known seasonal change of cardiovascular disease, which is also confirmed in our study, especially for the winter season, this study clearly evidenced the connection between daily changes in atmospheric parameters, temperatures, and atmospheric pressure, with ACS, its other clinical characteristics, and medical statuses of patients.

The results of our study show that the association between cold weather and temperature change with cases of ACS tends to have statistical significance in young patients probably because they are more exposed to atmospheric conditions. However, likely, the elderly population will also be affected by ACS, during cold weather conditions accompanied by changes in temperatures, due to lower thermoregulatory responses. A recent study demonstrated that the largest number of hospital admissions for ACS, for elderly patients occurred in the cold season [16].

Few studies have investigated a correlation between warm weather and ACS. In our study, there was a lower number of patients with ACS during the warm season. There was no observed association between high temperatures and ACS, although we evidenced a slight increase in ACS cases in the summer months, with an average temperature of 26 °C. In one of the largest meta-analyses, there was clear evidence for a positive relationship between increasing temperature and coronary heart disease (with a 2.8% increased risk for coronary disease for every 1 °C increase in temperature above reference temperatures) [25]. The data of our study also support the conclusion of a large study conducted in Vietnam [26], which emphasizes the role of extremely high and extremely low temperatures in increasing the incidence of ACS. Another extensive study emphasizes the role of very high temperatures, in the incidence of ACS, mainly when they are combined with very high values ​​of atmospheric pressure [27]. Another study [28] found positive associations between same-day apparent temperature and ischemic heart disease (% excess risk per 10 °F = 1.7 (95%CI = 0.2-3.3).

Regarding the link between ACS and temperature changes, our findings are consistent with previous studies that suggested a positive significant correlation of ACS with temperature changes [2,29,30].

Most studies have shown a higher incidence of ACS associated only with temperature factors. The impact of atmospheric pressure on cardiovascular diseases, especially on ACS, has been studied less frequently. Most studies analyzed only monthly or seasonal variations of event rates that are weaker than daily variations and often lead to inconclusive results.

Our study, in contrast, has shown complex effects, based on interactions of atmospheric parameters through simultaneous fluctuations in temperature and atmospheric pressure. We found a higher number of cases of ACS in those atmospheric conditions, where the difference from 48 hours before ACS and the day of occurrence of ACS in temperature was ≥4 °C and in pressure was ≥ 4 Pa.

One of the results of Danet et al.'s [6] study noted an increase in the incidence of ACS not only caused by a decrease in atmospheric pressure but also an increase in it. This progress was presented graphically in a U-shaped form, where an increase in atmospheric pressure is preceded one day before by a decrease in pressure.

A study conducted at Scott & White Memorial Hospital, Texas, showed a significant correlation (p = 0.0083) between a decrease in atmospheric pressure and the occurrence of ACS, one day after the decrease in pressure, especially during the autumn seasons and winter [18].

This observation remains consistent with our findings because our data predicted an increase in ACS in the 24 hours after the pressure drop, a time when the pressure often rises. Statistically significant effects of atmospheric pressure change, Pa24, and PaDayMI, are observed for the ≤ 44 age group and the 45-54 age group.

This relationship was most evident during the fall and winter months, which coincided with periods of greatest atmospheric pressure change in our study. In our study, it seems that there is a cause-effect relationship between the change in atmospheric pressure and the occurrence of ACS. The change in atmospheric pressure, more evident in the autumn and winter periods, precedes the appearance of the acute coronary event, based on the abovementioned mechanisms of mechanical stress on the atherosclerotic plaque. However, not every ACS is associated with changes in atmospheric pressure, and only certain plaques may be affected by changes in atmospheric pressure; however, explanations cannot be ruled out. Thus, it is difficult to specify the mechanisms of destabilization of the atherosclerotic plaque for each patient, but the multifactorial approach is more correct, giving the possibility that the prevention of ACS will be as efficient as possible.

Our study confirms that there is a significant association between atmospheric changes in interaction with clinical situations such as hypertension and diabetes. Based on our study, these patients have a higher risk of cardiovascular disease during specific atmospheric conditions because they have a higher atmospheric sensitivity. Among vulnerable people, these atmospheric conditions can be defined as cardiovascular risk factors with pathophysiological changes that directly promote the onset of ischemia.

People with meteoropathy who make up a large percentage of the population are individuals who respond to changes in meteorological conditions with a disease or worsening of a disease existing.

Limitations of the study

The study data were collected retrospectively using the data of registers and clinical charts, and not by interviewing the patients themselves, affecting the quality of the data. Only cases diagnosed with ACS, who underwent coronary angiography, were included in the study earlier. It would have been ideal to have included all cases with the diagnosis of CAD. Being a unicentric study may be another limitation of our study. Our study used available data of atmospheric parameters in nature, while there were no available data regarding the conditions of the internal atmosphere of the house, its environment work, etc. We used in our analysis only hypertension and diabetes, as risk factors for CAD and not the other factors for CAD. The study did not consider other components of atmospheric conditions, such as humidity, wind, solar radiation, or atmospheric pollution. Albania is a Mediterranean area, with mild winters and without drastic temperature changes and atmospheric pressure from day to day that could have different impacts on ACS.

## Conclusions

There is a significant relationship between seasonal changes in temperature and pressure atmospheric with ACS, where the autumn season prevails, followed by of spring, summer, and winter seasons. Significant changes in average monthly values ​​of temperature and pressure atmospheric conditions are accompanied by a significant increase in ACS cases, as happened in the months of March-April and October-November. Most of the cases in the cold period of the year (November-March) occurred in the days since there have been changes in temperature or atmospheric pressure. A significant relationship between the overall ACS admissions and the temperature 48 hours before the ACS was found. Meanwhile, specifically in patients with hypertension, there is a statistically significant relationship between the effect of temperature 48 hours before admission and the atmospheric pressure 48 hours before admission with the daily ACS cases. There is also a significant relationship in patients with diabetes mellitus between the effects of temperature 48 hours before admission and the atmospheric pressure 24 hours before and on the day of admission with the daily ACS cases.
